# HybridDBRpred: improved sequence-based prediction of DNA-binding amino acids using annotations from structured complexes and disordered proteins

**DOI:** 10.1093/nar/gkad1131

**Published:** 2023-12-04

**Authors:** Jian Zhang, Sushmita Basu, Lukasz Kurgan

**Affiliations:** School of Computer and Information Technology, Xinyang Normal University, Xinyang 464000, PR China; Department of Computer Science, Virginia Commonwealth University, Richmond, VA 23284, USA; Department of Computer Science, Virginia Commonwealth University, Richmond, VA 23284, USA

## Abstract

Current predictors of DNA-binding residues (DBRs) from protein sequences belong to two distinct groups, those trained on binding annotations extracted from structured protein-DNA complexes (structure-trained) vs. intrinsically disordered proteins (disorder-trained). We complete the first empirical analysis of predictive performance across the structure- and disorder-annotated proteins for a representative collection of ten predictors. Majority of the structure-trained tools perform well on the structure-annotated proteins while doing relatively poorly on the disorder-annotated proteins, and vice versa. Several methods make accurate predictions for the structure-annotated proteins or the disorder-annotated proteins, but none performs highly accurately for both annotation types. Moreover, most predictors make excessive cross-predictions for the disorder-annotated proteins, where residues that interact with non-DNA ligand types are predicted as DBRs. Motivated by these results, we design, validate and deploy an innovative meta-model, hybridDBRpred, that uses deep transformer network to combine predictions generated by three best current predictors. HybridDBRpred provides accurate predictions and low levels of cross-predictions across the two annotation types, and is statistically more accurate than each of the ten tools and baseline meta-predictors that rely on averaging and logistic regression. We deploy hybridDBRpred as a convenient web server at http://biomine.cs.vcu.edu/servers/hybridDBRpred/ and provide the corresponding source code at https://github.com/jianzhang-xynu/hybridDBRpred.

## Graphical Abstract

**Graphical Abstract:**
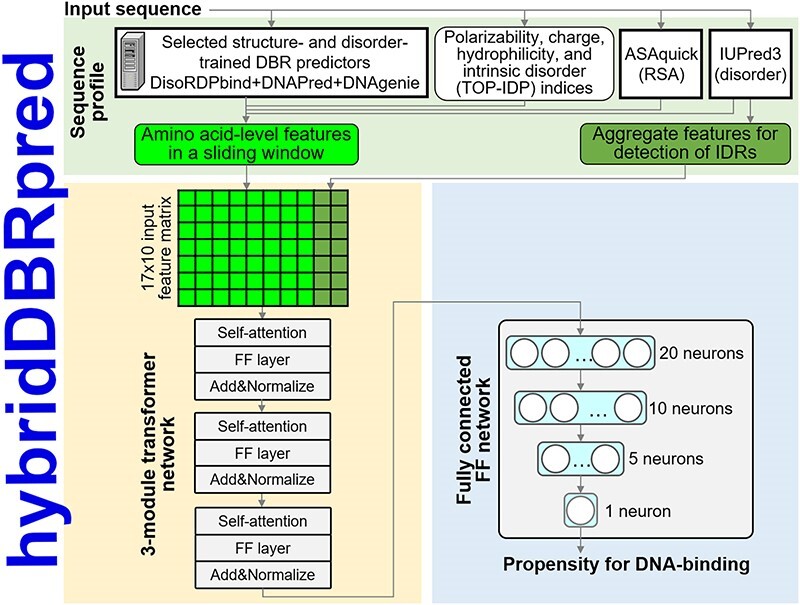

## Introduction

Protein-DNA interactions are central for many cellular functions including transcription, gene regulation, DNA repair, and chromatin remodelling (1,2). They are annotated and studied using a variety of experimental methods, such as affinity purification, electrophoresis mobility shift assays, chromatin immunoprecipitation, CRISPR-Cas9-based techniques and atomic force microscopy (3–5). Molecular-level details are learned using X-ray crystallography, electron microscopy, and nuclear magnetic resonance, with around 7000 structures of protein-DNA complexes in PDB (6). However, these techniques do not keep up with a rapid accumulation of the protein and DNA sequence data (7,8), motivating the development and use of fast computational predictors of protein-DNA interactions from protein sequences (9–14) and DNA sequences (15,16). These methods are developed using a limited amount of the experimental data and can be applied to predict interactions in a high-throughput manner for the uncharacterized sequences. The DNA sequence-based predictors were summarized and compared in a recent survey (15), while similar analysis is lacking for the protein sequence-based tools.

The protein sequence-based predictors include protein-level and residue-level tools. The protein-level methods predict whether a target protein sequence interacts with DNA. A few examples of recently released tools that predict DNA-binding proteins include StackDPPred (17), iDRBP_MMC (18), TargetDBP (19,20), DeepTFactor (21), RF-SVM (22), CNN-Pred (23) and TPSO-DBP (24). We focus on the residue-level tools that identify DNA binding residues (DBRs) in a given protein sequence since they provide more detailed information compared to the protein-level approaches. Using past surveys on this topic (9–14) and a manual literature search, we identified a comprehensive collection of 34 sequence-based predictors of DBRs. In chronological order, they include DBS-pred (25), DBS-PSSM (26), BindN (27), DNABindR (28), DP-bind (29,30), method by Ho *et al.* (31), DISIS (32), BindN-RF (33), DBindR (34), DBD-Threader (35), ProteDNA (36), BindN+ (37), NAPS (38), MetaDBSite (39), DNABR (40), TargetS (41), SNBRFinder (42), DisoRDPbind (43,44), DQPred-DBR (45), Dang *et al.* (46), TargetDNA (47), PRODNA (48), DRNApred (49), PDRLGB (50), ENSEMBLE-CNN (51), method by Zhang *et al.* (52), NucBind (53), hybridNAP (11), DNAPred (54), ProNA2020 (55), NCBRPred (56), MTDsite (57), DNAgenie (58) and DeepDISOBind (59). Some of these tools provide a broader scope by predicting DNA binding and RNA binding residues (BindN (27), BindN+ (37), NAPS (38), DRNApred (49), NucBind (53) and NCBRPred (56)); DNA, RNA and protein binding residues (hybridNAP (11), ProNA2020 (55), DisoRDPbind (43) and DeepDISOBind (59)); and DNA, RNA, carbohydrate and peptides binding residues (MTDsite (57)). Moreover, TargetS predicts residues that interact with 12 types of ligands including DNA, nucleotides, metal ions and the heme group. Virtually all of these methods utilize machine learning algorithms to derive their models. The early predictors rely on simple algorithms, such as Naïve Bayes (DNABindR) and shallow neural networks (DBS-pred and DBS-PSSM). Subsequent tools use more sophisticated algorithms including support vector machines (DQPred, ProteDNA, TargetDNA, DP-bind, DISIS, ProNA2020, NucBind, BindN, BindN+, SNBRFinder, TargetS, NCBRPred and DNAPred), decision trees and forests (NAPS, PDRLGB, DNABR, BindN-RF, and DBindR), and logistic regression (DisoRDPbind, DRNApred, and hybridNAP). Majority of the newest methods apply deep neural networks (MTDsite, DeepDISOBind, NCBRPred and ENSEMBLE-CNN). A common theme is that they train their models from training datasets using a process that minimizes the difference between their predictions and the known ground truth annotations. The trained models are then applied to predict DBRs for sequences outside the training datasets.

Based on their training datasets, they can be divided into two distinct groups: structure-trained predictors versus intrinsic disorder-trained predictors. The former group uses training datasets where annotations of DBRs are extracted from structures of protein–DNA complexes, typically using data from the PDB (6,60) and the PDB-derived BioLip (61,62) databases. The latter group utilizes training datasets collected from the DisProt database (63), where DBRs are located in the intrinsically disordered regions (IDRs). IDRs are segments in a protein sequence that do not have a stable three-dimensional structure under physiological conditions (64–66), which are especially abundant in eukaryotes (67,68). DBRs in IDRs are different from structured DBRs in several ways. They can interact with several different ligands by folding into different conformations, are enriched in disorder-promoting amino acids and have larger surface area (69–71). Furthermore, the importance of intrinsic disorder in the context of protein-DNA interactions was demonstrated in numerous studies (72–75). We identify two intrinsic disorder-trained predictors of DBRs, DisoRDPbind and DeepDISObind. This low number can be explained by the fact that the corresponding experimental annotations were introduced relatively recently (76). The remaining 32 methods are structure-trained and none of the 34 predictors are trained using annotations that span across structured and disordered states. The substantial differences between the structured and disordered states suggests that the current predictors may provide poor results for the other type of annotations. This claim is supported by recent studies that empirically found that structure-trained (disorder-trained) predictors of protein-binding residues and RNA-binding residues provide inaccurate predictions for the disorder-annotated (structure-annotated) proteins (77,78). However, the current structure-trained predictors of DBRs were never assessed on the disorder-annotated proteins and vice versa. Furthermore, recent works identify a cross-prediction problem where amino acids interacting with a given partner type are cross-predicted as interacting with different partner types, resulting in partner-agnostic predictions (49,53,56,77,79,80). In our case, this means that amino acids interacting with non-DNA partners (e.g. proteins and RNA) are predicted as DBRs. This may happen because sequence-based predictors of DBRs are typically trained on datasets composed of DNA-binding proteins, with few to no proteins that interact with the non-DNA partners. Thus, they might not be able to differentiate between different ligand types. While a few recent predictors of DBRs, including DRNApred, NCBRPred, and DisoRDPbind, were designed to reduce cross-predictions, a broad study that investigates this aspect is also missing.

Table 1 summarizes surveys that discuss predictors of protein–DNA interactions from protein sequences to examine whether literature already covers the above-mentioned aspects. The five reviews consider between 8 and 14 sequence-based predictors of DBRs and provide insightful information about their models, inputs and datasets that they utilize (9–14). Three reviews perform comparative analysis, but they cover a rather narrow subset of predictors of DBRs. This is because they focus on a broader spectrum of sequence- and structure-based predictors of DNA, RNA, and protein binding residues (10,11,13). Importantly, the five surveys do not discuss the disorder-trained predictors and recent methods that were published after 2018, do not evaluate the predictors on the disorder-annotated interactions, and do not investigate the cross-predictions. We study a substantially larger number of predictors, including nine recently published methods, and we address the open questions regarding predictive performance of the disorder-trained vs. structure-trained predictors. We empirically evaluate a representative set of ten sequence-based methods that include five structure-trained predictors of DBRs, both disorder-trained predictors of DBRs, and three disorder-trained methods that predict interactions with other partner types. We use a new test dataset that includes structure- and disorder-annotated proteins that share low similarity to the training datasets of the evaluated methods and some of which interact with the non-DNA partners, such as RNA and proteins. We use these annotations to comparatively assess the cross-predictions. Finally, driven by results of this analysis, we design, assess and release a new deep neural network-based meta-predictor, *hybridDBRpred* (*hybrid* network for *D*NA-*B*inding *R*esidue *pred*iction), that provides accurate predictions for the structure- and disorder-annotated proteins.

**Table 1.** Comparison of surveys that cover sequence-based predictors of DBRs

					Empirical evaluation			
Article	Predictor type covered	# sequence-based DBR predictors	Recent predictors (2019–2023)	Includes evaluation	# evaluated predictors	Covers structured & disordered data	Test data dissimilar from training data	Assesses cross-predictions
This survey	Structure- and disorder-trained	34	9	Yes	10	Yes	Yes	Yes
(11)	Structure-trained	9	0	Yes	3	No	No	No
(12)	Structure-trained	8	0	No	N/A	N/A	N/A	N/A
(10)	Structure-trained	14	0	Yes	5	No	Yes	No
(13)	Structure-trained	12	0	Yes	5	No	Yes	No
(14)	Structure-trained	12	0	No	N/A	N/A	N/A	N/A
(9)	Structure-trained	11	0	No	N/A	N/A	N/A	N/A

## Materials and methods

### Selection of predictors

We select a representative subset of the sequence-based predictors of DBRs. We focus on methods that are relatively recent, publicly available and sufficiently fast to predict a large dataset. More specifically, they must satisfy the following criteria: (i) published on or after 2010; (ii) the server or code was publicly available and functional when we collected predictions; (iii) output predictions for an average length sequence (300 amino acids) in <10 min; (iv) output real-valued propensities for DNA-binding and binary predictions for each amino acid, so their results can be evaluated with commonly used metrics. Consequently, we select five structure-trained methods: BindN+ (37), TargetS (41), TargetDNA (47), DNAPred (54) and DNAgenie (58); and both disorder-trained methods, DisoRDPbind (43) and DeepDISOBind (59). These methods include the two most recent predictors: the structure-trained DNAgenie and the disorder-trained DeepDISOBind. Using recent results from the CAID assessment (81), we supplement the disorder-trained DisoRDPbind and DeepDISOBind with three well-performing disorder-trained methods that satisfy the criteria and predict disordered binding residues, fMoRFpred (82), ANCHOR2 (83) and MoRFCHiBi (84). While these three methods were not originally designed to predict DBRs, we investigate whether they can be used for this purpose.

### Datasets

We train and test the hybridDBRpred method on datasets that cover structure-annotated and disorder-annotated proteins, and which include a sufficiently large number of residues that interact with the other/non-DNA ligand types to assess the cross-predictions. They include the training dataset that we use to train a machine-learning model, the validation dataset that we utilize to optimize predictive performance of this model, and the test dataset that we apply to compare performance with the current methods. We follow procedures from related studies to compile these datasets (80,85). Briefly, this means that we use full protein sequences where the binding annotations are mapped across different protein–DNA complexes that share the same protein into the same UniProt sequence using SIFTS (86), increasing their quality and completeness.

First, we collect the structure-based annotations of interactions from BioLip (62,87), which in turn processes data from PDB, and the disorder-based annotations from DisProt (63). Next, we cluster the collected proteins together with the combined set of training proteins for the 10 selected predictors (BindN+, TargetS, TargetDNA, DNAPred, DNAgenie, fMoRFpred, DisoRDPbind, ANCHOR2, MoRFCHiBi and DeepDISOBind) at 25% similarity using Blastclust (88). We pick one (the most recently released) protein from each cluster, which ensures that the selected proteins uniformly sample the sequence space and share low similarity. We select test proteins from the clusters that exclude any of the training proteins. Consequently, we collect 39 DNA-binding proteins and 396 proteins that interact with other ligand types, with the 2:1 rate of structure- vs. disorder- annotated proteins across both protein sets. The test dataset includes 435 proteins and 201 154 residues, with 2940 DBRs (1.5%) and 19 755 amino acids that interact with other ligand types (9.8%).

We source the training and validation datasets from clusters that include the training proteins of the 10 selected predictors and which exclude clusters used to pick the test proteins. This means that the training and validation proteins share low (<25%) similarity with the test proteins. We ensure that the validation dataset has similar numbers of the DNA-binding proteins when compared with the test dataset, which means that we select 13 disorder-annotated and 26 structure-annotated DNA-binding proteins into this dataset. We divide the remaining proteins proportionally between the training and validation dataset. Consequently, the training dataset has 591 proteins and 241 284 residues, with 4398 DBRs (1.82%) and 22 030 amino acids that interact with other ligand types (9.13%). The validation dataset includes 267 proteins and 116244 residues, with 2232 DBRs (1.92%) and 9960 amino acids that interact with other ligand types (8.57%). Supplementary Table S1 provides a detailed breakdown of the three datasets, which are freely available at http://biomine.cs.vcu.edu/servers/hybridDBRpred.

### Assessment metrics and statistical analysis

Evaluation is done at the residue-level and assesses the quality of the predicted real-valued propensities for DNA-binding and binary predictions. We evaluate propensities with the commonly used Area Under the ROC Curve (AUC). Moreover, motivated by the fact that DBRs constitute a small fraction of the residues (1.5%) and inspired by past studies (11,49,58,77,80), we also compute AULC (Area Under the Low false positive rate part of the ROC Curve). AULC quantifies AUC for arguably the most useful part of the curve where the number of predicted DBRs does not exceed the actual number of DBRs. Since AULC is a relatively small number, we compute AULCratio that divides AULC of a given method by the AULC of a random predictor. This way, AULCratio = 1 indicates a prediction that is equivalent to a random result while higher AULCratio quantifies the rate of improvement over the random result, e.g. AULCratio equals 2 when a given result is twice better than a random predictor. We evaluate the binary predictions (binds DNA versus does not bind DNA) using several complementary metrics (80):


\begin{eqnarray*} &&{\rm Sensitivity}= {\rm TPR} = {\rm TP}/({\rm TP} + {\rm FN})\\ &&{\rm FPR\ (false\ positive\ rate)} = 1 - {\rm Specificity} = {\rm FP}/({\rm TN} + {\rm FP})\\ &&{\rm F1} = 2({\rm TPR} \times (1 - {\rm FPR}))/({\rm TPR} + (1 - {\rm FPR}) \end{eqnarray*}

where TP, TN, FP and FN indicate the number of true positives (correctly predicted DBRs), true negatives (correctly predicted non-DBRs), false positives (non-DBRs incorrectly predicted as DBRs), and false negatives (DBRs incorrectly predicted non-DBRs), respectively. We derive binary predictions from the propensities using a threshold, where residues with propensities > threshold are assumed to bind DNA and the remaining residues are assumed not to bind. We standardize the thresholds across predictors to allow for reliable side-by-side comparisons. In particular, we use several thresholds to compare results for diverse predictive scenarios including low FPRs at 0.1 and 0.2 (given the low fraction of DBRs), and high sensitivities of 0.5 and 0.7.

Consistent with the assessments in several related studies (10,11,49), we evaluate false positives in two distinct categories, cross-predictions when they occur for residues that interact with the non-DNA ligands vs. over-predictions that we measure for residues that are not annotated to bind any ligands. Correspondingly, we compute two metrices: cross-prediction rate (CPR) =${\mathrm{\ }}F{P}_{non - DNA}$/${N}_{non - DNA}$, which quantifies the fraction of residues that bind non-DNA ligands that are predicted as DBRs, and over-prediction rate (OPR) =$F{P}_{non - binding}$/${N}_{non - binding}$, that is the fraction of non-binding residues predicted as DBRs among all non-binding residues. Similar to AULCratio, to ease interpretation of these values we report CPRratio and OPRratio that are computed as the CPR and OPR of a random predictor divided by the CPR and OPR of the evaluated method, respectively. This way, the values of the two ratios quantify the rate of improvement over the random result. We also assess the propensities using the area under the cross-prediction curve (AUCPC) and the area under the over-prediction curve (AUOPC), which analyze the relation between CPR and TPR, and between OPR and TPR, respectively. Importantly, higher AUOPC and AUCPC values mean that the amount of the over-predictions and cross predictions is higher/worse.

Lastly, we quantify statistical significance of differences between results produced by different predictors. This analysis finds whether one method provides consistently better results when compared with another tool over a broad range of different datasets. We perform 100 random selections of 20 DNA-binding and 40 non-DNA binding proteins, with equal split of the structure- and disorder-annotated proteins, from the benchmark dataset. We evaluate statistical significance of differences over these 100 paired results using the Student's *t*-test if the measurements are normal based on the Anderson–Darling test at 0.05 significance (89); otherwise we apply the Wilcoxon rank-sum test. We assume that the difference is significant if the resulting *P*-value < 0.01. This is consistent with recent related works (10,49,58,59,77).

### Architecture of the hybridDBRpred

Motivated by our empirical results that reveal that none of the current tools predicts accurately across the disorder-annotated and structure-annotated DNA-binding proteins, we design an innovative meta-predictor with the objective to significantly improve predictive performance. This meta-method utilizes a deep neural network to combine results generated by three complementary predictors of DBRs that include disorder-trained method (DisoRDPbind) and two structure-trained methods (DNAPred and DNAgenie). These methods produce accurate results for different proteins and different sequence regions (structure vs. disorder-trained), and so an effective way to combine their results requires identifying these differences using sequence-derived information. Consequently, we utilize three groups of inputs: (i) amino acid-level predictions of DBRs; (ii) amino acid-level hallmarks of DBRs that can be derived from the sequence (11), such as polarizability, charge, hydrophilicity, propensity for intrinsic disorder (90), solvent accessibility that we predict with the quick and accurate ASAquick (91), and putative intrinsic disorder that we generate using popular and fast IUPred3 and (iii) aggregate features that target detection of IDRs by calculating propensity for disorder for sequence segments. We detail these inputs in the Suppl. Table S2. Altogether, we introduce four innovations to generate accurate meta-predictions. In particular, we (i) design feature group 3 that facilitates detection of IDRs since disorder-trained methods are biased to perform better for the disorder-annotated proteins while structure-trained proteins tend to be more accurate for the structure-annotated proteins; (ii) use a sliding window to present the amino acid-level feature in groups 1 and 2 to the model, which provides useful context for the selection of the best input prediction of DBRs; (iii) utilize modern transformer modules to implement the deep neural network and (iv) train the transformer network using the binary cross-entropy loss function.

**Figure 1.**
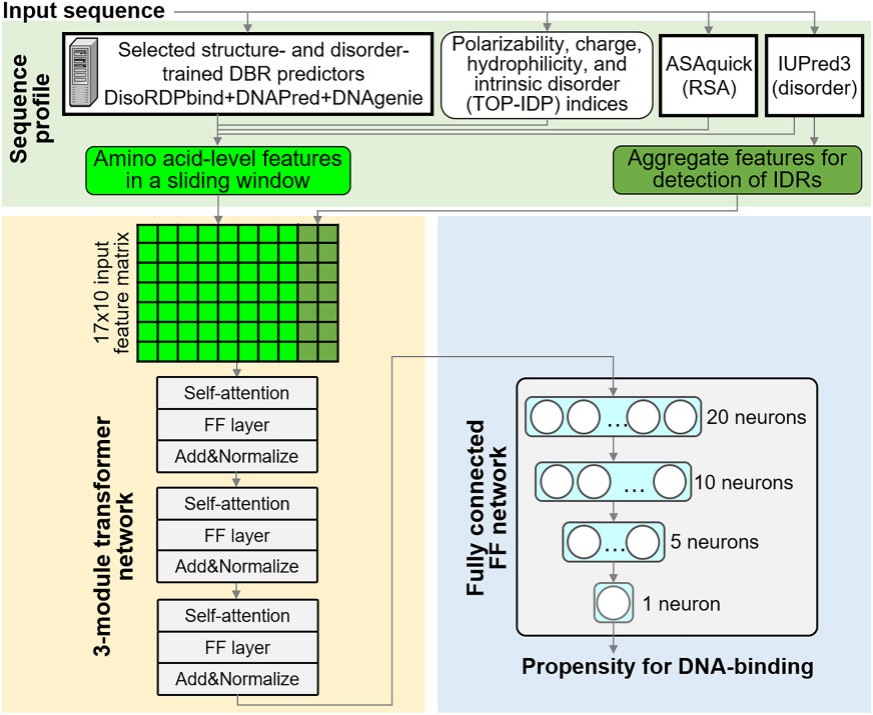
The topology of the hybridDBRpred predictor.

We summarize the architecture of our deep meta-predictor in Figure 1. First, we convert the protein sequence into the sequence profile. This profile includes the input groups 1 and 2, which total to 10 features that we process using a sliding window of size 15, and which we combine with 20 features from the input group 3 (light green box in Figure 1). Next, we feed the sequence profile into a deep transformer network (92) that consists of three stacked transformer modules (light yellow block in Figure 1). Each transformer includes a self-attention unit connected to a feedforward layer that is followed by a normalization layer before feeding into the subsequent transformer. We pass the normalized output of the last transformer to the fully connected feed forward network that we use to reduce the multidimensional latent space produced by the transformers into the predicted DNA-binding propensity (light blue box in Figure 1). The feed forward network gradually reduces the latent space from 20, to 10, to 5 and eventually to the one neuron which outputs the binding propensity. We train this architecture using Pytorch with the popular Adam optimizer and the binary cross-entropy loss function. We set the learning rate and batch size to 0.0001 and 128, respectively. We apply the binary cross-entropy loss function (93) instead of the default mean absolute error (L1), which is motivated by the use of the former function in several recent related studies (94–96). The binary cross-entropy loss function maximizes the likelihood of making correct predictions, penalizes incorrect predictions (large differences from the ground truth) more substantially than near-correct predictions, and converges faster.

## Results and discussion

### Comparative assessment of predictive performance

We primarily focus on investigating the ability of disorder-trained methods to make accurate predictions for the structure-annotated proteins and vice versa. The results are summarized in Table 2, with the corresponding ROC curves in Supplementary Figure S1A (entire benchmark dataset), S1D (disorder-annotated proteins in the benchmark dataset) and S1G (structure-annotated proteins in the benchmark dataset).

**Table 2.** Comparison of the ten structure-trained and disorder-trained predictors of binding residues and the new hybridDBRpred meta-predictor using the sampled test dataset

					Sensitivity		Specificity		
Dataset	Type of methods	Predictors	AUC	AULCratio at 0.1 FPR	at 0.1 FPR	at 0.2 FPR	at 0.5 TPR	at 0.7 TPR	maxF1
Structure-annotated proteins from the benchmark dataset									
	Structure-trained	TargetS	0.650 ± 0.026^+/+^	0.932 ± 0.407^+/+^	0.099 ± 0.033^+/+^	0.297 ± 0.054^+/+^	0.697 ± 0.035^+/+^	0.557 ± 0.034^+/+^	0.132 ± 0.012^+/+^
		TargetDNA	0.752 ± 0.027^+/+^	4.654 ± 0.763^+/+^	0.399 ± 0.050^+/+^	0.567 ± 0.050^+/+^	0.834 ± 0.031^+/+^	0.690 ± 0.044^+/+^	0.235 ± 0.028^+/+^
		BindN+	0.747 ± 0.018^+/+^	3.939 ± 0.534^+/+^	0.347 ± 0.032^+/+^	0.540 ± 0.034^+/+^	0.825 ± 0.020^+/+^	0.672 ± 0.029^+/+^	0.207 ± 0.020^+/+^
		DNAPred	0.808 ± 0.020 ^/+^	**7.314 ± 0.975** ^/–^	**0.509 ± 0.049 ^/=^**	0.649 ± 0.039 ^/+^	**0.904 ± 0.025 ^/=^**	0.756 ± 0.035 ^/+^	**0.335 ± 0.040 ^/–^**
		DNAgenie	0.748 ± 0.035^+/+^	3.476 ± 0.894^+/+^	0.325 ± 0.062^+/+^	0.546 ± 0.068^+/+^	0.821 ± 0.037^+/+^	0.676 ± 0.065^+/+^	0.205 ± 0.034^+/+^
	Disorder-trained	fMoRFpred	0.432 ± 0.018^+/+^	0.572 ± 0.145^+/+^	0.062 ± 0.010^+/+^	0.139 ± 0.014^+/+^	0.409 ± 0.024^+/+^	0.228 ± 0.023^+/+^	0.088 ± 0.010^+/+^
		ANCHOR2	0.468 ± 0.033^+/+^	0.171 ± 0.160^+/+^	0.029 ± 0.024^+/+^	0.127 ± 0.043^+/+^	0.453 ± 0.040^+/+^	0.306 ± 0.039^+/+^	0.092 ± 0.010^+/+^
		DeepDISObind	0.489 ± 0.035^+/+^	0.069 ± 0.083^+/+^	0.015 ± 0.015^+/+^	0.081 ± 0.035^+/+^	0.526 ± 0.045^+/+^	0.386 ± 0.047^+/+^	0.095 ± 0.012^+/+^
		MoRFchibi	0.533 ± 0.027^+/+^	1.293 ± 0.346^+/+^	0.121 ± 0.030^+/+^	0.216 ± 0.034^+/+^	0.545 ± 0.042^+/+^	0.358 ± 0.033^+/+^	0.097 ± 0.012^+/+^
		DisoRDPbind	0.621 ± 0.030^+/+^	1.965 ± 0.458^+/+^	0.187 ± 0.036^+/+^	0.333 ± 0.047^+/+^	0.657 ± 0.043^+/+^	0.475 ± 0.049^+/+^	0.127 ± 0.015^+/+^
	Baseline meta-predictors	Average-based	0.815 ± 0.017^–/+^	6.324 ± 0.736^+/–^	0.488 ± 0.045^+/+^	0.674 ± 0.037^–/+^	0.895 ± 0.017^+/=^	0.781 ± 0.030^–/+^	0.294 ± 0.030^–/=^
		Logistic regression	0.777 ± 0.019^+/+^	4.619 ± 0.857^+/+^	0.395 ± 0.051^+/+^	0.580 ± 0.047^+/+^	0.861 ± 0.023^+/+^	0.739 ± 0.034^+/+^	0.237 ± 0.025^+/+^
	Deep learning meta-predictor	hybridDBRpred	**0.827 ± 0.017^–/^**	5.704 ± 0.724^+/^	0.504 ± 0.042^=/^	**0.704 ± 0.040^–/^**	0.900 ± 0.016^=/^	**0.802 ± 0.028^–/^**	0.291 ± 0.027^+/^
Disorder- annotated proteins from the benchmark dataset									
	Structure-trained	TargetS	0.557 ± 0.030^+/+^	1.932 ± 0.291^+/+^	0.181 ± 0.026^+/+^	0.311 ± 0.036^+/+^	0.581 ± 0.048^+/+^	0.349 ± 0.047^+/+^	0.130 ± 0.016^+/+^
		TargetDNA	0.529 ± 0.030^+/+^	1.892 ± 0.362^+/+^	0.170 ± 0.030^+/+^	0.280 ± 0.037^+/+^	0.528 ± 0.048^+/+^	0.301 ± 0.040^+/+^	0.122 ± 0.018^+/+^
		BindN+	0.566 ± 0.034^+/+^	2.522 ± 0.562^+/+^	0.206 ± 0.018^+/+^	0.327 ± 0.045^+/+^	0.598 ± 0.058^+/+^	0.343 ± 0.047^+/+^	0.144 ± 0.025^+/+^
		DNAPred	0.535 ± 0.031^+/+^	2.342 ± 0.375^+/+^	0.187 ± 0.027^+/+^	0.281 ± 0.035^+/+^	0.557 ± 0.049^+/+^	0.298 ± 0.047^+/+^	0.130 ± 0.017^+/+^
		DNAgenie	0.683 ± 0.058 ^/+^	3.769 ± 1.144 ^/+^	0.323 ± 0.079 ^/+^	0.447 ± 0.094 ^/+^	0.753 ± 0.083 ^/+^	0.564 ± 0.092 ^/+^	0.220 ± 0.054 ^/+^
	Disorder-trained	fMoRFpred	0.512 ± 0.014^+/+^	1.313 ± 0.187^+/+^	0.118 ± 0.011^+/+^	0.204 ± 0.013^+/+^	0.488 ± 0.025^+/+^	0.321 ± 0.021^+/+^	0.107 ± 0.020^+/+^
		ANCHOR2	0.585 ± 0.056^+/+^	1.551 ± 0.634^+/+^	0.158 ± 0.053^+/+^	0.315 ± 0.078^+/+^	0.614 ± 0.083^+/+^	0.420 ± 0.089^+/+^	0.133 ± 0.023^+/+^
		DeepDISObind	0.640 ± 0.066^+/+^	1.799 ± 0.771^+/+^	0.117 ± 0.065^+/+^	0.331 ± 0.095^+/+^	0.670 ± 0.083^+/+^	0.499 ± 0.116^+/+^	0.147 ± 0.027^+/+^
		MoRFchibi	0.628 ± 0.028^+/+^	2.370 ± 0.491^+/+^	0.214 ± 0.038^+/+^	0.369 ± 0.040^+/+^	0.687 ± 0.043^+/+^	0.461 ± 0.041^+/+^	0.155 ± 0.031^+/+^
		DisoRDPbind	0.632 ± 0.028^+/+^	2.508 ± 0.486^+/+^	0.235 ± 0.035^+/+^	0.392 ± 0.042^+/+^	0.710 ± 0.044^+/+^	0.469 ± 0.050^+/+^	0.163 ± 0.021^+/+^
	Baseline meta-predictors	Average-based	0.655 ± 0.037^+/+^	3.610 ± 0.668^=/+^	0.302 ± 0.047^=/+^	0.469 ± 0.051^=/+^	0.769 ± 0.049^=/+^	0.485 ± 0.077^+/+^	0.200 ± 0.034^+/+^
		Logistic regression	0.678 ± 0.025^=/+^	3.129 ± 0.617^+/+^	0.274 ± 0.038^+/+^	0.411 ± 0.045^+/+^	0.756 ± 0.036^=/+^	0.584 ± 0.038^=/+^	0.186 ± 0.027^+/+^
	Deep learning meta-predictor	hybridDBRpred	**0.766 ± 0.048^–/^**	**4.805 ± 1.140^–/^**	**0.396 ± 0.079^–/^**	**0.569 ± 0.082^–/^**	**0.844 ± 0.048^–/^**	**0.723 ± 0.061^–/^**	**0.258 ± 0.055^–/^**
Entire benchmark dataset									
	Structure-trained	TargetS	0.593 ± 0.012^+/+^	1.549 ± 0.246^+/+^	0.147 ± 0.021^+/+^	0.287 ± 0.029^+/+^	0.641 ± 0.029^+/+^	0.455 ± 0.032^+/+^	0.120 ± 0.012^+/+^
		TargetDNA	0.631 ± 0.024^+/+^	3.034 ± 0.396^+/+^	0.268 ± 0.030^+/+^	0.403 ± 0.035^+/+^	0.708 ± 0.037^+/+^	0.454 ± 0.039^+/+^	0.169 ± 0.016^+/+^
		BindN+	0.644 ± 0.024^+/+^	3.085 ± 0.422^+/+^	0.267 ± 0.028^+/+^	0.422 ± 0.034^+/+^	0.729 ± 0.017^+/+^	0.337 ± 0.037^+/+^	0.170 ± 0.017^+/+^
		DNAPred	0.659 ± 0.025^+/+^	4.505 ± 0.531^+/+^	0.329 ± 0.032^=/+^	0.445 ± 0.035^+/+^	0.747 ± 0.034^+/+^	0.501 ± 0.049^+/+^	0.221 ± 0.020^+/+^
		DNAgenie	0.703 ± 0.036 ^/+^	3.595 ± 0.714 ^/+^	0.327 ± 0.047 ^/+^	0.503 ± 0.059 ^/+^	0.795 ± 0.057 ^/+^	0.585 ± 0.062 ^/+^	0.208 ± 0.032 ^/+^
	Disorder-trained	fMoRFpred	0.477 ± 0.013^+/+^	1.006 ± 0.137^+/+^	0.092 ± 0.010^+/+^	0.176 ± 0.012^+/+^	0.457 ± 0.019^+/+^	0.282 ± 0.015^+/+^	0.094 ± 0.012^+/+^
		ANCHOR2	0.534 ± 0.032^+/+^	1.431 ± 0.450^+/+^	0.147 ± 0.037^+/+^	0.233 ± 0.044^+/+^	0.535 ± 0.053^+/+^	0.337 ± 0.037^+/+^	0.108 ± 0.014^+/+^
		DeepDISObind	0.566 ± 0.032^+/+^	1.309 ± 0.445^+/+^	0.132 ± 0.039^+/+^	0.261 ± 0.061^+/+^	0.565 ± 0.056^+/+^	0.415 ± 0.036^+/+^	0.115 ± 0.016^+/+^
		MoRFchibi	0.585 ± 0.022^+/+^	1.775 ± 0.310^+/+^	0.163 ± 0.023^+/+^	0.298 ± 0.030^+/+^	0.621 ± 0.033^+/+^	0.411 ± 0.029^+/+^	0.119 ± 0.016^+/+^
		DisoRDPbind	0.626 ± 0.021^+/+^	2.282 ± 0.323^+/+^	0.214 ± 0.025^+/+^	0.364 ± 0.031^+/+^	0.694 ± 0.034^+/+^	0.470 ± 0.036^+/+^	0.143 ± 0.014^+/+^
	Baseline meta-predictors	Average-based	0.726 ± 0.023^–/+^	4.839 ± 0.485^–/+^	0.387 ± 0.032^–/+^	0.560 ± 0.035^–/+^	0.840 ± 0.021^–/+^	0.644 ± 0.047^–/+^	0.239 ± 0.021^–/+^
		Logistic regression	0.720 ± 0.016^–/+^	3.747 ± 0.513^+/+^	0.328 ± 0.031^=/+^	0.485 ± 0.034^=/+^	0.812 ± 0.020^=/+^	0.642 ± 0.028^–/+^	0.207 ± 0.019^=/+^
	Deep learning meta-predictor	hybridDBRpred	**0.786 ± 0.023^–/^**	**5.187 ± 0.485** ^–/^	**0.432 ± 0.036^–/^**	**0.619 ± 0.036^–/^**	**0.874 ± 0.016^–/^**	**0.727 ± 0.035^–/^**	**0.262 ± 0.024^–/^**

We report averages and the corresponding standard deviations over the 100 subsets (see ‘Assessment metrics and statistical analysis’ section for details). We provide sensitivity that is calibrated for all methods to the same FPR = 0.1 and 0.2, and specificity calibrated to the sensitivity = TPR = 0.5 and 0.7; This allows for a direct comparison between methods under several diverse predictive scenarios. The best results for a given dataset and for each column are in bold font. We report results from the statistical significance test using superscript in the ‘x/y’ format where x indicates comparison against the current method with the highest AUC and y stands for the comparison against the new hybridDBRpred meta-predictor; +, =, and – denote that the best current predictor or hybridDBRpred is significantly better, not significantly different, significantly worse than another method, respectively, at *P*-value < 0.01.

For the structure-annotated proteins, Table 2 shows that four of the five structure-trained methods produce predictions with AUC > 0.74 and that DNAPred achieves the highest AUC = 0.81. These are relatively accurate predictions, as suggested by the AULCratio values that range between 3.5 and 7.3 for these four tools. On the other hand, the disorder-trained methods provide low-quality results, with AULCratios ranging between 0.07 and 1.9, and DisoRDPbind producing the highest AUC of 0.62. We observe the same trend when using binary metrics. For instance, the four structure-trained methods obtain sensitivity values at the 0.1 FPR between 0.32 and 0.51, while the best disorder-trained DisoRDPbind has sensitivity = 0.19. While the poor performance of ANCHOR2, fMoRFpred and MoRFchibi can be attributed to the fact that they were trained to predict disordered residues that bind to proteins and peptides, DisoRDPbind and DeepDISObind target prediction of DBRs and still perform rather poorly. This likely stems from the fact that their predictive models that are trained from the disorder-annotated proteins do not generalize into the structure-annotated protein–DNA interactions.

For the disorder-annotated proteins, we find that the two disorder-trained predictors of DBRs outperform most of the structure-trained methods, securing AUCs of 0.64 (DeepDISObind) and 0.63 (DisoRDPbind). The one exception is the structure-trained DNAgenie that has AUC of 0.68 and AULCratio of 3.8, and outperforms the disorder-trained methods. DNAgenie is a recently published tool that utilizes a training dataset of DNA-binding proteins collected from PDB, which are processed to map data from multiple protein–DNA complexes onto the same protein, resulting in a more complete set of binding annotations. It also uses disorder predictions as an input, which facilitates identifying putative disordered binding residues that undergo disorder-to-order transitions upon binding DNA (97). These factors can explain DNAgenie's ability to produce good results for the disorder-annotated proteins. Overall, we find that while the disorder-trained methods perform relatively well for the disorder-annotated proteins, the best results are secured by DNAgenie. These observations partly agree with the related recent studies of predictions of protein-binding and RNA-binding residues (77,78). While we similarly show that disorder-trained methods are outperformed by the structure-trained tools for the structure-annotated proteins, we find that structure-trained DNAgenie performs favorably on the disorder-annotated proteins. However, DNAgenie and the disorder-trained methods are outperformed by the structure-trained DNAPred on the structure-annotated proteins. This means that a single predictor does not provide consistently best results for both structure-annotated and disorder-annotated proteins.

The evaluation on the full benchmark dataset provides further details. We find that the best results are produced by the structure-trained DNAgenie (AUC of 0.70 and AULCratio of 3.6) and DNAPred (AUC of 0.66 and AULCratio of 4.5). Their predictions are statistically significantly better than the results of the other eight methods (*P*-value < 0.01). The best disorder-trained DisoRDPbind obtains AUC = 0.63 and AULCratio = 2.3. The corresponding ROC curves are in the Supplementary Figure S1A. DNAgenie performs modestly well on the structure-annotated proteins (third-best AUC) and very well on the disorder-annotated proteins (best AUC, statistically better than all other methods at *P*-value < 0.01). DNAPred is the best option for the structure-annotated proteins (best AUC, statistically better than all other methods at *P*-value < 0.01) but performs poorly for the disorder-annotated proteins. DisoRDPbind has the third-best AUC for the disorder-annotated proteins and performs by far the best among the disorder-trained tools for the structure-annotated proteins. The low-quality results produced by the disorder-trained ANCHOR2, fMoRFpred and MoRFchibi are due to the fact that these tools predict disordered protein and peptide binding residues. The modest performance of the structure-trained TargetS likely comes from the broad scope of this model, which predicts 12 different types of ligands.

To summarize, we find that none of the current tools offers results that are the most accurate across the two types of annotations. The best results for the structure-annotated proteins are offered by the structure-trained DNAPred, for the disorder-annotated proteins by the structure-trained DNAgenie, and DisoRDPbind is the best disorder-trained tool.

### Analysis of the cross-predictions and over-predictions

Recent studies demonstrate that some sequence-based predictors of binding residues suffer high cross-prediction rates, which means that they essentially predict binding residues in a ligand-agnostic manner (49,53,56,58,77,79,80,85). Table 3 analyses the false positives generated by the ten predictors to quantify the cross-prediction errors (residues that bind non-DNA ligands predicted as DBRs) and over-prediction errors (non-binding residues predicted as DBRs). The corresponding cross-prediction curves and over-prediction curves are in the Supplementary Figure S1. The AUCPC (area under the cross-prediction curve) and AUOPC (area under the over-prediction curve) of 0.5 correspond to random levels of performance while lower values indicate lower amounts of the cross- and over-predictions. On the other hand, CPRratio and OPRratio quantify the rate of improvement over a random predictor, where higher values denote more accurate results. TargetS, fMoRFpred, ANCHOR2 and MoRFchibi produce high amounts of cross-predictions and over-prediction with AUCPC >0.4 and/or AUOPC >0.4. This can be explained by the fact that TargetS was designed to predict interactions with multiple ligand types and since fMoRFpred, ANCHOR2 and MoRFchibi predict disordered residues that bind proteins and peptides. This means that the cross-predictions are expected for these methods. We focus our analysis on the other six predictors.

**Table 3.** The evaluation of cross-predictions and over-predictions for the 10 structure-trained and disorder-trained predictors of binding residues and the new hybridDBRpred meta-predictor using the sampled test dataset

Dataset	Type of methods	Predictors	CPRratio at 0.1 FPR	AUCPC	OPRratio at 0.1 FPR	AUOPC
Structure-annotated proteins from benchmark dataset						
	Structure-trained	TargetS	0.404 ± 0.125^+/+^	0.570 ± 0.033^+/+^	1.062 ± 0.354^+/+^	0.341 ± 0.027^+/+^
		TargetDNA	2.431 ± 0.406^+/+^	0.328 ± 0.032^+/+^	4.129 ± 0.531^+/+^	0.245 ± 0.027^+/+^
		BindN+	2.218 ± 0.457^+/+^	0.325 ± 0.034^+/+^	3.587 ± 0.332^+/+^	0.250 ± 0.018^+/+^
		DNAPred	2.988 ± 0.525 ^/+^	0.276 ± 0.030 ^/+^	**5.319 ± 0.540 ^/^** ^=^	0.180 ± 0.020 ^/+^
		DNAgenie	2.140 ± 0.781^+/+^	0.330 ± 0.065^+/+^	3.355 ± 0.633^+/+^	0.249 ± 0.035^+/+^
	Disorder-trained	fMoRFpred	0.785 ± 0.180^+/+^	0.556 ± 0.023^+/+^	0.624 ± 0.105^+/+^	0.569 ± 0.018^+/+^
		ANCHOR2	1.531 ± 1.306^+/+^	0.441 ± 0.063^+/+^	0.288 ± 0.243^+/+^	0.536 ± 0.033^+/+^
		DeepDISObind	1.013 ± 0.904^+/+^	0.404 ± 0.052^+/+^	0.149 ± 0.149^+/+^	0.515 ± 0.035^+/+^
		MoRFchibi	1.199 ± 0.383^+/+^	0.462 ± 0.039^+/+^	1.240 ± 0.308^+/+^	0.467 ± 0.027^+/+^
		DisoRDPbind	2.420 ± 0.671^+/+^	0.353 ± 0.036^+/+^	1.879 ± 0.367^+/+^	0.380 ± 0.031^+/+^
	Baseline meta-predictors	Average-based	3.178 ± 0.781^=/+^	0.254 ± 0.033^–/+^	5.033 ± 0.486^–/=^	0.182 ± 0.017^=/+^
		Logistic regression	3.259 ± 0.747^–/+^	0.274 ± 0.027^=/+^	4.151 ± 0.548^+/+^	0.218 ± 0.019^+/+^
	Deep learning meta-predictor	hybridDBRpred	**4.004 ± 1.207^–/^**	**0.210 ± 0.038^–/^**	5.156 ± 0.442^=/^	**0.172 ± 0.017^–/^**
Disorder- annotated proteins from benchmark dataset						
	Structure-trained	TargetS	1.447 ± 0.414^+/+^	0.463 ± 0.046^+/+^	1.927 ± 0.259^+/+^	0.440 ± 0.029^+/+^
		TargetDNA	1.204 ± 0.342^+/+^	0.541 ± 0.037^+/+^	1.882 ± 0.359^+/+^	0.458 ± 0.031^+/+^
		BindN+	1.591 ± 0.505^+/+^	0.475 ± 0.046^+/+^	2.240 ± 0.426^+/+^	0.427 ± 0.035^+/+^
		DNAPred	1.586 ± 0.447^+/+^	0.496 ± 0.040^+/+^	1.999 ± 0.289^+/+^	0.459 ± 0.031^+/+^
		DNAgenie	3.077 ± 1.268 ^/+^	0.323 ± 0.071 ^/+^	3.415 ± 0.877 ^/+^	0.316 ± 0.058 ^/+^
	Disorder-trained	fMoRFpred	0.947 ± 0.158^+/+^	0.515 ± 0.020^+/+^	1.255 ± 0.128^+/+^	0.483 ± 0.016^+/+^
		ANCHOR2	1.044 ± 0.568^+/+^	0.571 ± 0.076^+/+^	1.874 ± 0.675^+/+^	0.386 ± 0.057^+/+^
		DeepDISObind	2.615 ± 3.071^+/+^	0.454 ± 0.085^+/+^	1.794 ± 0.689^+/+^	0.342 ± 0.067^+/+^
		MoRFchibi	1.615 ± 0.515^+/+^	0.428 ± 0.043^+/+^	2.347 ± 0.395^+/+^	0.362 ± 0.027^+/+^
		DisoRDPbind	2.037 ± 0.587^+/+^	0.389 ± 0.046^+/+^	2.491 ± 0.398^+/+^	0.364 ± 0.028^+/+^
	Baseline meta-predictors	Average-based	2.618 ± 0.843^+/+^	0.371 ± 0.051^+/+^	3.200 ± 0.526^=/+^	0.341 ± 0.037^+/+^
		Logistic regression	2.111 ± 0.734^+/+^	0.354 ± 0.052^+/+^	3.110 ± 0.457^+/+^	0.309 ± 0.026^=/+^
	Deep learning meta-predictor	hybridDBRpred	**4.864 ± 2.327^–/^**	**0.237 ± 0.064^–/^**	**3.979 ± 0.883^–/^**	**0.234 ± 0.049^–/^**
Entire from benchmark dataset						
	Structure-trained	TargetS	0.952 ± 0.207^+/+^	0.469 ± 0.040^+/+^	1.586 ± 0.236^+/+^	0.401 ± 0.022^+/+^
		TargetDNA	1.771 ± 0.366^+/+^	0.448 ± 0.032^+/+^	2.875 ± 0.340^+/+^	0.361 ± 0.024^+/+^
		BindN+	1.926 ± 0.477^+/+^	0.400 ± 0.038^+/+^	2.827 ± 0.291^+/+^	0.351 ± 0.024^+/+^
		DNAPred	2.472 ± 0.533^+/+^	0.387 ± 0.035^+/+^	3.452 ± 0.338^+/+^	0.336 ± 0.025^+/+^
		DNAgenie	2.988 ± 1.003 ^/+^	0.287 ± 0.053 ^/+^	3.349 ± 0.483 ^/+^	0.298 ± 0.036 ^/+^
	Disorder-trained	fMoRFpred	0.765 ± 0.137^+/+^	0.548 ± 0.018^+/+^	0.956 ± 0.103^+/+^	0.520 ± 0.014^+/+^
		ANCHOR2	0.660 ± 0.257^+/+^	0.630 ± 0.046^+/+^	1.754 ± 0.444^+/+^	0.447 ± 0.033^+/+^
		DeepDISObind	1.379 ± 0.762^+/+^	0.578 ± 0.047^+/+^	1.357 ± 0.422^+/+^	0.417 ± 0.033^+/+^
		MoRFchibi	1.424 ± 0.427^+/+^	0.439 ± 0.035^+/+^	1.687 ± 0.224^+/+^	0.413 ± 0.021^+/+^
		DisoRDPbind	1.844 ± 0.428^+/+^	0.395 ± 0.037^+/+^	2.212 ± 0.269^+/+^	0.371 ± 0.021^+/+^
	Baseline meta-predictors	Average-based	3.242 ± 0.864^–/+^	0.293 ± 0.038^=/+^	4.008 ± 0.338^–/+^	0.271 ± 0.022^–/+^
		Logistic regression	2.516 ± 0.772^+/+^	0.315 ± 0.042^+/+^	3.552 ± 0.350^–/+^	0.272 ± 0.017^–/+^
	Deep learning meta-predictor	hybridDBRpred	**5.413 ± 1.918^–/^**	**0.201 ± 0.042^–/^**	**4.275 ± 0.380^–/^**	**0.216 ± 0.023^–/^**

We report averages and the corresponding standard deviations over the 100 subsets (see ‘Assessment metrics and statistical analysis’ section for details). The best results for a given dataset and for each column are shown in bold font. We report results from the statistical significance test using superscript in the ‘x/y’ format where x indicates comparison against the current method with the highest AUC and y stands for the comparison against the new hybridDBRpred meta-predictor; +, =, and – denote that the best current predictor or hybridDBRpred is significantly better, not significantly different, significantly worse than another method at *P*-value < 0.01.

For the structure-annotated proteins, DeepDISObind performs poorly with AUCPC and AUOPC > 0.4, which means that it substantially overpredicts DBRs. The remaining methods perform relatively well with AUOPC on average at about 0.25 and AUCPC on average at about 0.33. The best structure-trained method, DNAPred, secures AUCPC = 0.276 and AUOPC = 0.180, demonstrating a good ability to selectively and accurately predict DBRs for the structure-annotated proteins. The same trends are reflected by the CPRratio and OPRratio scores, where DNAPred obtains the best results and high values of 3.0 and 5.3, respectively. The structure-trained methods (TargetDNA, BindN+, DNAPred, and DNAgenie) perform better than the disorder-trained DisoRDPbind, which is expected for this protein set. Moreover, the cross-prediction rates are higher than the over-predictions rates (i.e. OPRratios > CPRratios), which suggests that these methods are biased to cross-predict between the ligand types.

For the disorder-annotated proteins, the structure-trained TargetDNA, BindN+, and DNAPred perform poorly with AUCPC and AUOPC > 0.4. The disorder-trained DeepDISObind also makes substantial amounts of cross-predictions (AUCPC = 0.45). The only two well-performing methods are the structure-trained DNAgenie and the disorder-trained DisoRDPbind. They obtain AUCPC and AUOPC at around 0.32 (DNAgenie) and 0.37 (DisoRDPbind), and are the only tools with CPRratio and OPRratio > 2.

Using the full benchmark dataset, we find that DeepDISObind and TargetDNA suffer high rates of cross-predictions and over-predictions. Supplementary Figure S1B and S1C plot the corresponding cross-prediction and the over-prediction curves. DNAgenie is the best tool that secures AUCPC = 0.29, AUOPC = 0.30, CPRratio = 3.0 and OPRratio = 3.3, which suggests that it is at least three times better than a random predictor. These results are also statistically better than the results of the other nine methods (*P*-value < 0.01). DNAPred, BindN+, and DisoRDPbind perform reasonably well, with AUCPC and AUOPC ≤0.4 and CPRratio and OPRratio at around or over 2.

To sum up, the structure-trained DNAgenie produces overall the best, low and balanced amounts of cross-predictions and over-predictions. The structure-trained DNAPred is better than DNAgenie for the structure-annotated proteins but makes excessively large amounts of cross-predictions for the disorder-annotated proteins. The best disorder-trained DisoRDPbind generates modest amounts of both cross- and over-predictions that are similar across the structure- and disorder-annotated proteins. The other tools make large amounts of errors, particularly in terms of the cross-predictions for the disorder-annotated proteins. Their AUCPC values are around 0.5, which suggests that they effectively predict all binding residues, irrespective of the ligand type.

### Comparative assessment of hybridDBRpred's predictive performance

Our analysis reveals that none of the ten methods provides the most accurate results across both structure- and disorder-annotated proteins. Some methods perform well on the disorder-annotated proteins (DNAgenie and DisoRDPbind) or the structure-annotated proteins (DNAPred, TargetDNA, and BindN+). Moreover, some methods suffer relatively high cross-prediction rates. These findings are in line with the results of recent works that investigated predictors of protein-binding and RNA-binding residues and which developed new meta-predictors that overcome their limitations (77,78). Motivated by these studies and the trade-offs that we uncovered, we investigate whether a meta-predictor that combines well-performing structure-trained and intrinsic disorder-trained predictors of DBRs would provide significant improvements in the predictive performance. Our hybridDBRpred meta-predictor relies on a modern deep transformer network and sequence-derived inputs that provide useful context to accurately combine predictions from the three arguably best current tools: DisoRDPbind, DNAPred and DNAgenie.

Table 2 compares hybridDBRpred against the ten predictors. We find that hybridDBRpred produces the best predictions on the full benchmark dataset, with AUC = 0.786, AULCratio = 5.19, F1 = 0.26 and sensitivity = 0.43 at 0.1 FPR. These results are statistically higher than the predictions of the current methods (*p*-value ≤ 0.01). To compare, the best scores generated by the current methods are AUC = 0.703 (DNAgenie), AULCratio = 4.50 (DNAPred), F1 = 0.22 (DNAPred), and sensitivity = 0.33 at 0.1 FPR (DNAPred). More importantly, hybridDBRpred generates accurate predictions for both the structure-annotated and the disorder-annotated proteins. It obtains the highest AUC = 0.827 for the structure-annotated proteins and also the highest AUC = 0.766 for the disorder-annotated proteins. Moreover, hybridDBRpred produces low amounts of the cross-prediction and over-prediction errors. Table 3 reveals that hybridDBRpred's AUCPC that quantifies the level of cross-predictions is 0.201 for the entire test dataset, 0.210 for the structure-annotated test proteins, and 0.237 for the disorder-annotated test proteins, compared to the best (lowest) AUCPC values of the current tools that are 0.287 (DNAgenie), 0.276 (DNAPred), and 0.323 (DNAgenie), respectively. Similarly, the hybridDBRpred's over-predictions that we quantify with AUOPC are 0.216 on the full test set, 0.172 for the structure-trained proteins, and 0.234 for the disorder-trained proteins. These are all better (lower) than the results of the existing tools that secure the best AUOPCs of 0.298 (DNAgenie), 0.180 (DNAPred), and 0.316 (DNAgenie), respectively. The improvements in AUCPC and AUOPC values when contrasting hybridDBRpred against each of the ten methods are statistically significant (*P*-value ≤ 0.01). In short, the new deep learning-based hybridDBRpred meta-predictor substantially improves over the ten current tools, provides balanced quality of predictions for the disorder-annotated and structure-annotated proteins, and generates low levels of cross-predictions.

We also compare hybridDBRpred with two baseline meta-predictors. The baselines include a logistic regression model that utilizes the same inputs as hybridDBRpred and a simple meta-predictor that computes average of the propensities produced by the three tools that we use as hybridDBRpred's inputs. We detail these baselines in the supplement. Table 2 shows that the baselines secure similar levels of predictive performance with the AUC of 0.720 (average-based) and 0.726 (regression) on the entire test set, compared to the statistically better AUC of 0.786 from hybridDBRpred (*P*-value < 0.01). Our deep transformer-based solution also provides statistically significant improvements in AUC for both the structure-annotated and the disorder-annotated test proteins (*P*-value < 0.01; Table 2). Furthermore, hybridDBRpred outperforms both baselines in the context of the cross-predictions and over-predictions. It obtains AUCPC of 0.201 vs. 0.293 and 0.315 for the baselines, and AUOPC of 0.216 versus 0.271 and 0.272 for the baselines; these differences are statistically significant (*P*-value < 0.01; Table 3). These results reveal that the deep transformer-based model provides large improvements over simpler meta-predictors. Next, we investigate contributions of specific elements of our model to these improvements.

### Ablation analysis

The hybridDBRpred meta-predictor relies on four key innovations: calculation of aggregate features for detection of IDRs; application of the sliding window to collect inputs; use of the transformer modules to design the deep network; and training with the binary cross-entropy loss function. We perform ablation analysis by removing one of these innovations at the time and measuring the difference in the predictive performance when compared to the complete hybridDBRpred model. Supplementary Figure S2 summarizes these results on the test dataset by considering the overall predictive performance measured with AUC and the amount of cross-predictions and over-predictions quantified with AUCPC and AUOPC, respectively. We find that the removal of each of the four innovations leads to a statistically significant drop in predictive quality measured with each of the three metrics (*P*-values < 0.01). The features for the detection of IDRs contribute the most to the reduction of cross-predictions (Supplementary Figure S2B). The use of the binary cross-entropy loss function helps with improving both cross-predictions and over-predictions (Supplementary Figure S2B and C). The inclusion of the transformer modules and the sliding window has substantial impact on the overall predictive quality and also reduces the cross- and over-predictions (Supplementary Figure S2A–C). Altogether, these results suggest that each of the four innovation contributes to the favorable predictive performance of our deep meta-predictor.

We also investigate contributions of each of the three input predictors of DBRs to our meta-predictor. Supplementary Figure S3 compares results of the complete hybridDBRpred model with the three versions of that model where one of the input predictions is removed. This ablation experiment demonstrates that the removal of any of the three inputs produces a statistically significant drop in the predictive quality measured with AUC, AUCPC and AUOPC (*P*-values < 0.01). In particular, the AUC decreases from 0.786 (the complete hybridDBRpred model) to 0.754, 0.748, and 0.737 when DisoDRPbind, DNAPred and DNAgenie is excluded, respectively. While these values are still better than the AUCs of 0.726 and 0.720 for the baseline meta-predictors (Table 2), which is due to the use of the above-mentioned innovations, they demonstrate that the inclusion of each of the three predictions in hybridDBRpred is warranted.

### HybridDBRpred web server

The hybridDBRpred method is freely available as a convenient web server at http://biomine.cs.vcu.edu/servers/hybridDBRpred/. It requires only the FASTA-formatted protein sequence as input. It automates the entire prediction process on the server side by running predictions by DNAPred, DisoRDPbind and DNAgenie, generating the inputs to the deep learner, and processing predictions using the transformer network. The server takes about 2 min to predict an average size sequence with about 200 residues. Upon completion of the prediction, users can browse the color-coded prediction results on the webpage and receive text-formatted results to the email address that they (optionally) provide. The outputs produced by the server include the putative propensities from DNAPred, DNAgenie, and DisoRDPbind, together with the predictions of hybridDBRpred. We archive the results for at least three months.

### Analysis of the putative DNA-binding residues

Native annotations of DBRs, particularly for the structure-annotated proteins, rely on somehow subjective protocols. Most of the methods assume that a given residue binds DNA if at least one of its atoms is close enough to one of the DNA’s atoms, using a few different distance thresholds including 3.5 Å (25,34,37,49) and 4.5Å (36). BioLiP, which was used to annotate data for DNAgenie, applies a more sophisticated approach where the distance is computed as 0.5 Å plus the sum of the Van der Waal's radii of the closest protein atom and DNA atom (87). These differences may result in different annotations of native DBRs for the same protein.

**Figure 2.**
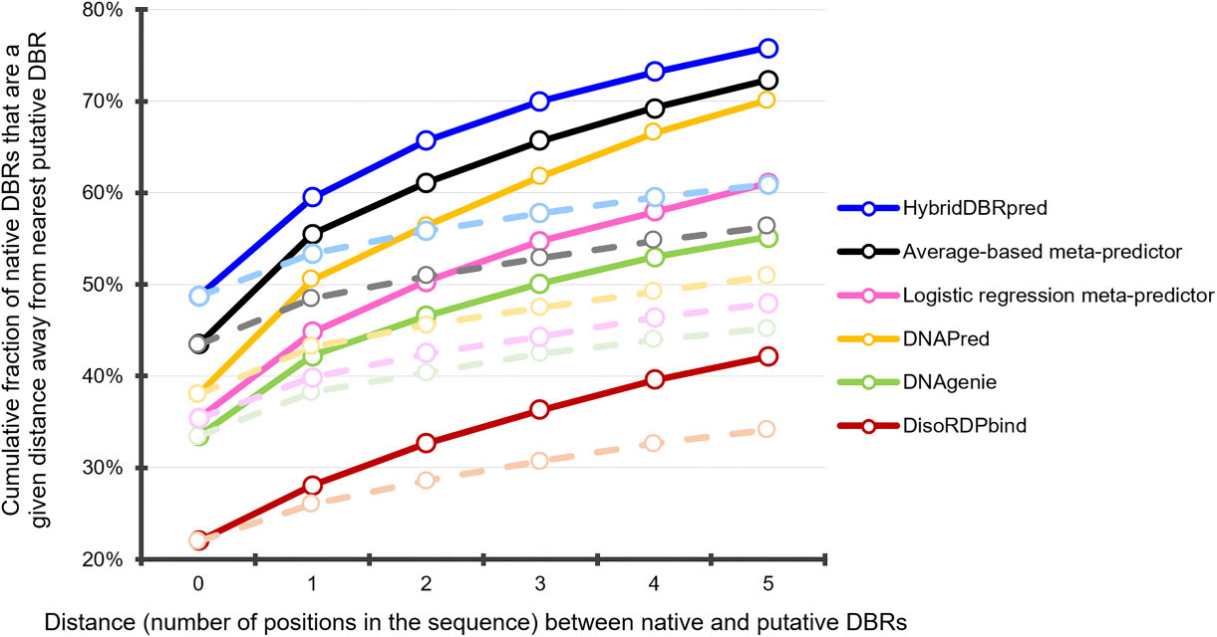
TPR values (*y*-axis) computed in the function of the number of positions in the sequence between the evaluated residues and the nearest native DBRs (*x*-axis). We consider the four best performing methods from Table 2 and perform this test on the test dataset. We compute the TPR values by assuming that putative DBRs that are within a given number of positions away from the native DBR are correct. Solid lines report results based on the predictions from the four methods while the color-coded dotted lines represent corresponding baselines where DNA-binding residues are predicted at random in the same proportions as the putative binding residues generated by the predictors.

We study whether predictions from the best performing methods, including DNAPred, DNAgenie, DisoRDPbind, the two baseline meta-predictors, and hybridDBRpred, are sensitive to these differences by investigating whether the false positives (incorrectly predicted DBRs) are biased to localize close to the native DBRs. Figure 2 analyses the presence of putative DBRs nearby the native DBRs in the sequence; we cannot perform this analysis using proximity in the structure since some annotations concern disordered regions. The *x*-axis quantifies the number of positions between the residues that we analyze and the nearest native DBRs, while the *y*-axis gives TPR values when assuming that the putative DBRs within the distance defined by the *x*-axis are correct. In other words, we count predicted DBRs that are within *x* = {1, 2, 3, 4, 5} positions from the native DBRs as true positives (solid lines in Figure 2). We compare these results against baselines where DNA-binding residues are predicted at random in the same proportions as the putative binding residues generated by the considered predictors (dotted lines in Figure 2 that are color-coded for the corresponding predictors). We find that disproportionally higher numbers of putative DBRs are located immediately adjacent to a native DBR. This can be measured by comparing the increase in TPRs between *x* = 0 and *x* = 1 and the subsequent positions with the corresponding baseline results. For instance, TPR of hybridDBRpred grows from 0.49 to 0.59 ((0.59–0.49)/0.49 = 20% increase) between *x* of 0 and 1, and further to 0.66 between *x* of 1 and 2 ((0.66–0.59)/0.59 = 12% increase). The corresponding baseline grows from 0.49 for *x* = 0 to 0.53 for *x* = 1 (8% increase), and to 0.56 for *x* = 2 (6% increase). We observe higher growth for lower values of *x* for the predictions and substantially larger values when comparing predictions against the baselines. These observations are true for all other methods (Figure 2), e.g. 0.12 (*x* = 0 to *x* = 1) vs. 0.06 (*x* = 1 to *x* = 3) increases for DNAPred; 0.09 vs. 0.04 for DNAgenie; 0.12 vs. 0.06 for the average-based baseline meta-predictor; they are all coupled with substantially lower values for the baselines. Overall, Figure 2 shows diminishing slopes for each of the six curves that reveals that false positives are concentrated around the positions of the native DBRs. This implies that DBRs predicted for the amino acids adjacent to the native DBRs in the sequence could be driven by the threshold-dependent nature of annotations, and perhaps should not be treated as mistakes. That suggests that the predictive performance that we calculate for these tools might underestimate their actual performance. These findings agree with recent studies that similarly identify an increase in the ‘false positives’ near the positions of native protein- and nucleic acids-binding residues (49,85).

## Summary and conclusions

Current sequence-based predictors of DBRs belong to two distinct groups, those trained on the structure-annotated proteins vs. the disorder-annotated proteins. We identify and summarize a comprehensive collection of 34 predictors. We select a representative set of 10 predictors, which include 7 predictors of DBRs and 3 predictors of disordered binding regions. We use them to perform a first-of-its-kind empirical analysis of their ability to accurately predict DBRs using novel and low-similarity benchmark dataset composed of the structure-annotated and the disorder-annotated proteins. The most accurate predictions for the structure-annotated proteins are offered by the structure-trained predictors, including the best DNAPred, while the disorder-trained methods perform poorly for these proteins. Moreover, the structure-trained DNAgenie performs well for the disorder-annotated proteins and DisoRDPbind is the best disorder-trained tool. These observations complement results of recent studies that focus on the evaluation of the predictions of protein-binding and RNA-binding residues (77,78). Analysis of false positives reveals that they are disproportionally concentrated in the vicinity of the native DBRs. This likely stems from a somewhat arbitrary nature of the native annotations of DBRs and suggests that we could be underestimating the actual predictive performance. Moreover, we suggest that more accurate disorder-trained tools are needed due to modest levels of predictive performance of the current tools. We also study the cross-predictions, where residues that bind other/non-DNA ligand types are predicted as DBRs. Except for DNAgenie and DisoRDPbind, the other considered methods make excessive amounts of cross predictions for the disorder-annotated proteins, effectively making ligand-agnostic predictions of all binding residues. Furthermore, we find that TargetDNA, BindN+, DNAPred, DNAgenie, and DisoRDPbind produce relatively low amount of cross-predictions for the structure-annotated proteins.

Most importantly, our empirical results suggest that none of the considered tools offer predictions that are highly accurate across the disorder-annotated and structure-annotated proteins, motivating the development of a novel meta-predictor. We conceptualize, design, implement, empirically validate and deploy the hybridDBRpred meta-model that combines predictions generated by three arguably most accurate current predictors. Our solution uses a well-designed collection of sequence-derived features and the deep transformer network that we train with an advanced loss function to produce accurate predictions of DBRs. We demonstrate empirically that these innovative design choices produce substantial improvements to the predictive quality of hybridDBRpred. Overall, hybridDBRpred provides balanced and high levels of predictive quality across the two annotation types and generates relatively low levels of cross-predictions and over-predictions. We also show that our deep learning-based meta-predictor is statistically more accurate than the results produced by each of the ten tools as well as baseline meta-predictors that rely on simple averaging and logistic regression. We implement hybridDBRpred as a convenient web server that is freely available at http://biomine.cs.vcu.edu/servers/hybridDBRpred/. We also provide the corresponding source code at https://github.com/jianzhang-xynu/hybridDBRpred.

## Supplementary Material

gkad1131_supplemental_file

## Data Availability

hybridDBRpred is freely available at http://biomine.cs.vcu.edu/servers/hybridDBRpred/. The corresponding source code is available at https://github.com/jianzhang-xynu/hybridDBRpred and https://zenodo.org/doi/10.5281/zenodo.10081016.
