# Supplementary material for: HybridDBRpred: improved sequence-based prediction of DNA-binding amino acids using annotations from structured complexes and disordered proteins
Source: Nucleic Acids Res. 2023 Dec 4;52(2):e10. doi: 10.1093/nar/gkad1131 (PMC10810184; doi:10.1093/nar/gkad1131)
Supplement: gkad1131_supplemental_file [file gkad1131_supplemental_file.pdf]

Supplementary Materials for HybridDBRpred: improved sequence-based prediction of DNA-binding amino acids using annotations from structured complexes and disordered proteins

Jian Zhang [1,*], Sushmita Basu [2], Lukasz Kurgan [2,*]
[1] *School of Computer and Information Technology, Xinyang Normal University, Xinyang, 464000, P.R. China*
[2] *Department of Computer Science, Virginia Commonwealth University, Richmond, VA 23284, USA*
[*] *Corresponding authors: jianzhang@xynu.edu.cn; lkurgan@vcu.edu*

Baseline meta-predictors

Inspired by the meta-predictors designed in refs (1,2), we design two simple baseline meta-predictors. We use these baselines to evaluate the magnitude of improvements brought by devising the more sophisticated deep transformer-based meta-predictor.

The first baseline considers combining the min-max normalized scores generated by 5 tools that secure accurate results on the test dataset: TargetDNA, BindN+, DNApred, DNAgenie, and DisoRDPbind (Tables 2 and 3 in the main text). We use two approaches: an average, which uses all input predictions collectively to generate the result, and a maximum, which selects the highest propensity and corresponds to using a union of the input predictions. We use these two approaches when considering combinations of 2, 3, 4 and 5 out of the 5 predictors. We compare these results in Suppl. Table S3. The best single predictor secures AUC = 0.703 and AULCratio = 3.59. Combining results of two methods (DNAPred and DisoRDPbind) improves AUC to 0.717 and AULCratio to 3.82. Adding the third method into the meta-predictor (DNAPred, DisoRDPbind, and DNAgenie) again increases the predictive quality to AUC = 0.726 and AULCratio = 4.83. However, the meta-approaches that utilize four methods and five methods do not provide further improvements, and secure AUC = 0.720 and AULCratio = 4.91, and AUC = 0.709 and AULCratio = 4.79, respectively. This is because the methods that are being added (BindN+ and TargetDNA) are inferior to the three tools that were already included in the meta-predictor (Table 2 in the main text). The average-based consensus is more accurate than the maximum-based approach, irrespective of the number of input predictions used. This is because each of the input methods has weak elements which can be better alleviated by combining them together. The average-based ensemble of DNAPred, DisoRDPbind, and DNAgenie constitutes **the first baseline**.

The **second baseline** utilizes the same inputs as the transformer-based hybridDBRpred but relies on a simple logistic regression model, instead of the deep neural network. We pick the logistic regression motivated by the use of this machine learning algorithms in recent relates studies (3,4).

References

1. Zhang, J., Ghadermarzi, S. and Kurgan, L. (2020) Prediction of protein-binding residues: dichotomy of sequence-based methods developed using structured complexes versus disordered proteins. *Bioinformatics*, **36**, 4729-4738.
2. Zhang, F., Li, M., Zhang, J. and Kurgan, L. (2023) HybridRNAbind: prediction of RNA interacting residues across structure-annotated and disorder-annotated proteins. *Nucleic Acids Research*.
3. Zhang, J., Ghadermarzi, S., Katuwawala, A. and Kurgan, L. (2021) DNAgenie: accurate prediction of DNA-type-specific binding residues in protein sequences. *Briefings in Bioinformatics*, **22**, bbab336.
4. Zhang, J. and Kurgan, L. (2019) SCRIBER: accurate and partner type-specific prediction of protein-binding residues from proteins sequences. *Bioinformatics*, **35**, i343-i353.

Supplementary Tables

Supplementary Table S1. Summary of the benchmark dataset.

Dataset type	Annotation type	Number of proteins			Number of residues		
		All	DNA-binding	non-DNA-binding	All	DNA-binding	Other-ligand-binding
Training	All proteins	591	87	504	241,284	4,398 (1.8%)	22,030 (9.1%)
	Disorder-annotated proteins	197	29	168	84,506	2,810 (3.3%)	14,841 (17.6%)
	Structure-annotated proteins	394	58	336	156,778	1,588 (1.0%)	7,189 (4.6%)
Validation	All proteins	267	39	228	116,244	2,232 (1.9%)	9,960 (8.6%)
	Disorder-annotated proteins	89	13	76	40,764	1,527 (3.7%)	6,370 (15.6%)
	Structure-annotated proteins	178	26	152	75,480	705 (0.9%)	3,590 (4.8%)
Test	All proteins	435	39	396	201,154	2,940 (1.5%)	19,755 (9.8%)
	Disorder-annotated proteins	145	13	132	75,643	1,133 (1.5%)	13,768 (18.2%)
	Structure-annotated proteins	290	26	264	125,511	1,807 (1.4%)	5,987 (4.7%)

Supplementary Table S2. Description of features that utilized in the hybridDBRpred model.

Input group	Description	Number of features
Amino acid-level predictions of DBRs	Predicted DNA-binding propensities from DisoRDPbind	1
	Predicted DNA-binding propensities from DNAPred	1
	Predicted DNA-binding propensities from DNAgenie	1
Amino acid-level hallmarks of DBRs	Polarizability, charge, hydrophilicity, and intrinsic disorder (TOP-IDP index)	4
	Solvent accessibility predicted from sequence with ASAquick	1
	Intrinsic disorder propensities for long IDRs and short IDRs predicted from sequence with IUPred3	2
Aggregate features for detection of IDRs computed for sequence window	Average and standard deviation of the intrinsic disorder propensities for long IDRs and short IDRs predicted from sequence with IUPred3	$2 \times 2 = 4$
	Putative disorder content (% of disordered residues) computed from the intrinsic disorder propensities for long IDRs and short IDRs predicted from sequence with IUPred3 that are binarized with four thresholds = {0.4, 0.5, 0.6, 0.7}	$2 \times 4 = 8$
	Length of the longest putative IDR computed from the intrinsic disorder propensities for long IDRs and short IDRs predicted from sequence with IUPred3 that are binarized with four thresholds = {0.4, 0.5, 0.6, 0.7}	$2 \times 4 = 8$

Supplementary Table S3. Predictive performance of the simple/baseline meta-predictors that combine results of TargetDNA, BindN+, DNApred, DNAgenie, and DisoRDPbind using the test dataset. We report average and the corresponding standard deviations over the 100 subsets (details in the "Assessment metrics and statistical analysis" section in the main text). We compare the results of the best predictor, DNAgenie, with the best combination of the 2, 3, 4 and all 5 predictors that is computed using the average and maximum of their min-max normalized propensities.

Number methods	Operator used: best combination of methods	AUC	AULCratio	Sensitivity		Specificity		maxF1	CPRratio	AUCPC	OPRratio	AUOPC
				at 0.1 FPR	at 0.2 FPR	at 0.5 TPR	at 0.7 TPR					
Best	N/A: DNAgenie	0.703±0.036	3.595±0.714	0.327±0.047	0.503±0.059	0.795±0.057	0.585±0.062	0.208±0.032	2.988±1.003	**0.287±0.053**	3.349±0.483	0.298±0.036
Best 2	Max: DNApred DNAgenie	0.709±0.022	4.523±0.573	0.365±0.034	0.523±0.030	0.819±0.024	0.609±0.040	0.229±0.025	**3.315±0.899**	0.316±0.036	3.744±0.389	0.288±0.021
	Avg: DNApred disoRDPbind	0.717±0.029	3.819±0.578	0.329±0.044	0.512±0.045	0.808±0.031	0.636±0.052	0.208±0.028	3.020±0.975	0.293±0.045	3.368±0.448	0.282±0.028
Best 3	Max: DNApred DNAgenie DisoRDPbind	0.711±0.021	4.245±0.562	0.351±0.036	0.526±0.031	0.820±0.022	0.603±0.042	0.220±0.024	3.230±0.903	0.317±0.035	3.588±0.395	0.286±0.021
	Avg: DNApred DNAgenie DisoRDPbind	**0.726±0.023**	**4.839±0.485**	0.387±0.032	**0.560±0.035**	**0.840±0.021**	**0.644±0.047**	**0.239±0.021**	3.242±0.864	0.293±0.038	**4.008±0.338**	**0.271±0.022**
Best 4	Max: BindN+ DNApred DNAgenie DisoRDPbind	0.701±0.022	4.226±0.550	0.346±0.034	0.521±0.030	0.816±0.022	0.594±0.047	0.218±0.023	3.073±0.793	0.331±0.035	3.549±0.379	0.296±0.021
	Avg: BindN+ DNApred DNAgenie DisoRDPbind	0.720±0.022	4.909±0.475	**0.388±0.030**	0.555±0.033	0.837±0.020	0.630±0.050	0.241±0.019	2.983±0.719	0.314±0.037	4.059±0.306	0.277±0.022
All five:	Max: the five predictors	0.696±0.022	3.902±0.511	0.328±0.035	0.510±0.032	0.807±0.022	0.599±0.044	0.206±0.021	2.620±0.613	0.349±0.033	3.406±0.383	0.300±0.021
	Avg: the five predictors	0.709±0.022	4.785±0.470	0.378±0.030	0.533±0.033	0.825±0.023	0.612±0.050	0.236±0.018	2.722±0.602	0.338±0.035	3.993±0.314	0.286±0.022

Supplementary Figures

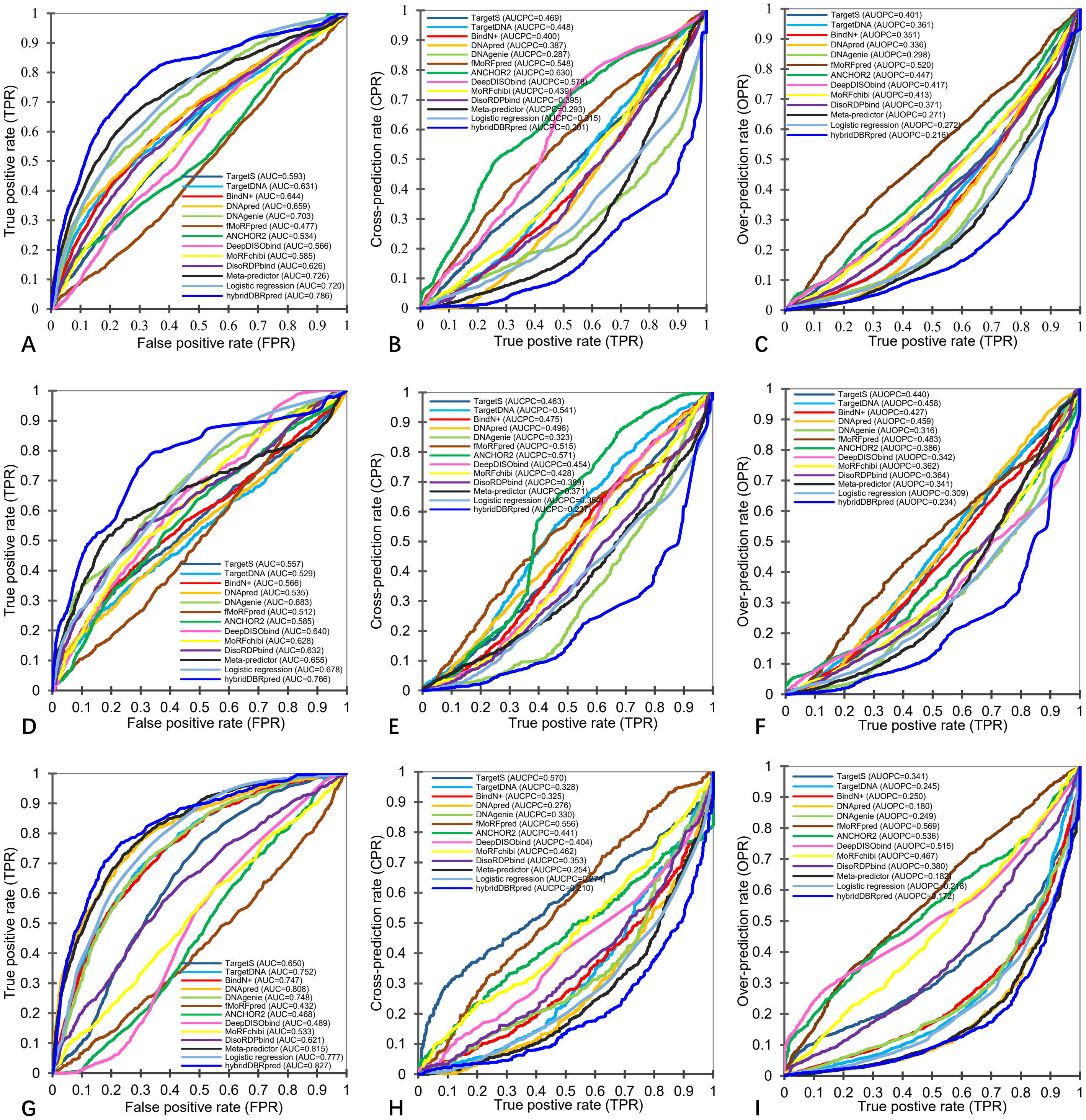

Supplementary Figure S1. The ROC curves (panel A), cross-prediction curves (panel B), and the over-prediction curves (panel C) computed on the test dataset. The ROC curves (panel D), cross-prediction curves (panel E), and the over-prediction curves (panel F) computed on the disorder-annotated proteins in the test dataset. The ROC curves (panel G), cross-prediction curves (panel H), and the over-prediction curves (panel I) computed on the structure-annotated proteins in the test dataset.

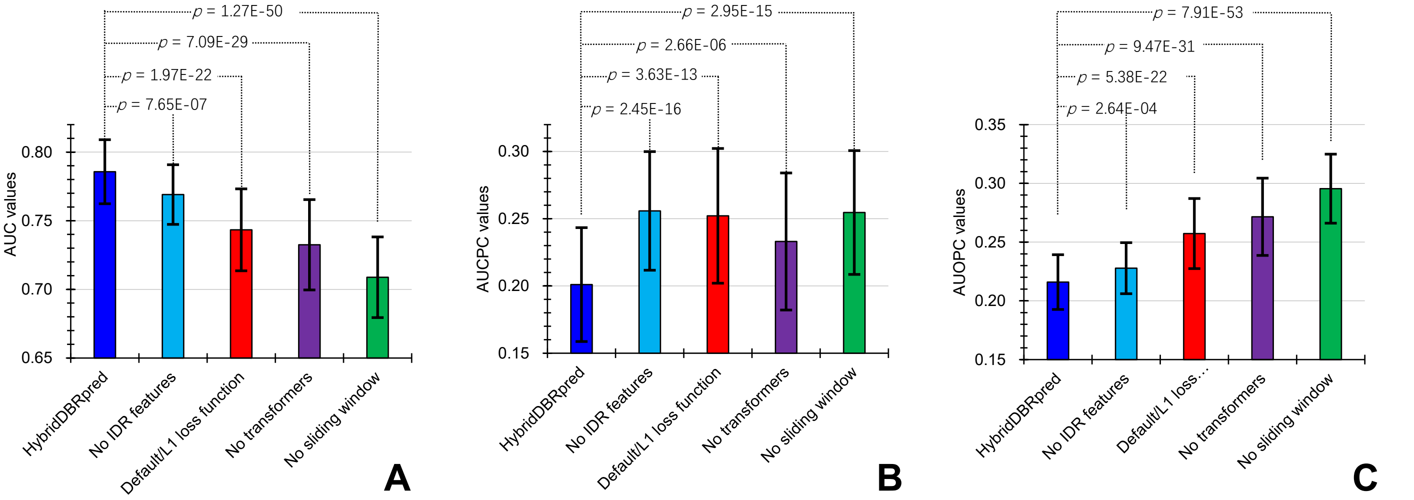

Supplementary Figure S2. Comparison of the predictive performance on the test dataset for hybridDBRpred and four versions of this model where one of the four innovations was removed. The performance is quantified with AUC values that measure overall performance (panel A; higher values are better), AUCPC that measure the amount of cross predictions (panel B; lower values are better), and AUOPC that measure the extend of the over predictions (panel C; lower values are better). We report averages (bars), the corresponding standard deviations (error bars) and the *p*-values (top of the panel) that were generated by comparing against the complete hybridDBRpred model over the 100 subsets of the test dataset (see "Assessment metrics and statistical analysis" section in the main text for details).

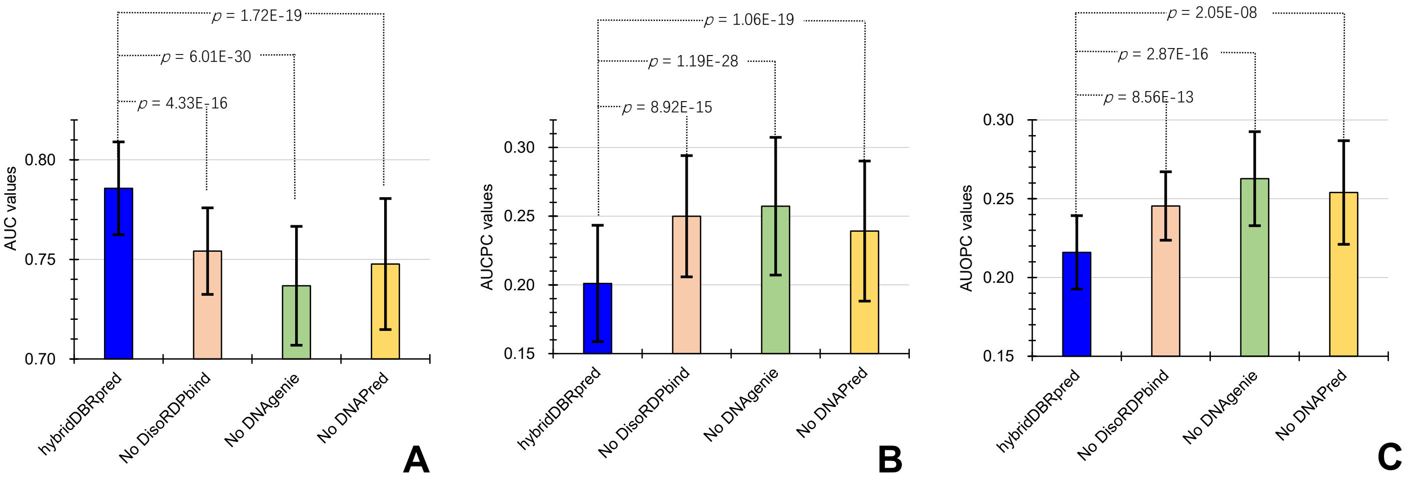

Supplementary Figure S3. Comparison of the predictive performance on the test dataset for hybridDBRpred and three versions of this model where one of the three input predictions of DBRs was excluded. The performance is quantified with AUC values that measure overall performance (panel A; higher values are better), AUCPC that measure the amount of cross predictions (panel B; lower values are better), and AUOPC that measure the extend of the over predictions (panel C; lower values are better). We report averages (bars), the corresponding standard deviations (error bars) and the *p*-values (top of the panel) that were generated by comparing against the complete hybridDBRpred model over the 100 subsets of the test dataset (see "Assessment metrics and statistical analysis" section in the main text for details).
